# Overall survival with maintenance olaparib in platinum-sensitive relapsed ovarian cancer by somatic or germline BRCA and homologous recombination repair mutation status

**DOI:** 10.1038/s41416-025-02966-x

**Published:** 2025-03-17

**Authors:** Sandro Pignata, Amit Oza, Geoff Hall, Beatriz Pardo, Radoslaw Madry, David Cibula, Jaroslav Klat, Ana Montes, Rosalind Glasspool, Nicoletta Colombo, Imre Pete, Ana Herrero Ibáñez, Margarita Romeo, Rumyana Ilieva, Constanta Timcheva, Massimo Di Maio, Zahid Bashir, Rosie Taylor, Alan Barnicle, Andrew Clamp

**Affiliations:** 1 Department of Urology and Gynecology, Istituto Nazionale Tumori ‘Fondazione G Pascale’, IRCCS, Napoli, Italy; 2 Division of Medical Oncology and Hematology, Princess Margaret Cancer Centre, Toronto, ON Canada; 3 Leeds Institute of Medical Research, St James’s University Hospital, Leeds, UK; 4 Department of Medical Oncology, ICO l’Hospitalet – Hospital Duran i Reynals, IDIBELL, L’Hospitalet de Llobregat, Barcelona, Spain; 5 Department of Gynecologic Oncology, Medical University Karol Marcinkowski, Poznań, Poland; 6 Department of Obstetrics and Gynaecology, First Faculty of Medicine, Charles University and General University Hospital in Prague, Prague, Czech Republic; 7 Department of Obstetrics and Gynecology, University Hospital Ostrava, and University of Ostrava, Ostrava Poruba, Czech Republic; 8 Department of Oncology, Cancer Centre, Guy’s and St Thomas’ NHS Foundation Trust, London, UK; 9 Medical Oncology Department, Beatson West of Scotland Cancer Centre and University of Glasgow, Glasgow, UK; 10 Department of Medicine and Surgery, University of Milan-Bicocca and European Institute of Oncology IRCCS, Milan, Italy; 11 Department of Gynecology, National Institute of Cancer, Budapest, Hungary; 12 Servico de Oncología Médica, Hospital Universitario Miguel Servet, Zaragoza, Spain; 13 Medical Oncology Department, ICO Badalona – Hospital Universitari Germans Trias i Pujol, Badalona, Spain; 14 Medical Oncology Clinic, MHAT “Central Onco Hospital”, OOD, Plovdiv, Bulgaria; 15 Medical Oncology Clinic, MHAT for Women’s Health – Nadezhda, OOD, Sofia, Bulgaria; 16 Department of Oncology, University of Turin, at Mauriziano Hospital, Turin, Italy; 17 Global Medical Affairs, AstraZeneca, Cambridge, UK; 18 GMA Payer Biometrics, Oncology R&D, AstraZeneca, Cambridge, UK; 19 Translational Medicine, Oncology R&D, AstraZeneca, Cambridge, UK; 20 Department of Medical Oncology, The Christie NHS Foundation Trust and University of Manchester, Manchester, UK

**Keywords:** Medical research, Oncology

## Abstract

**Background:**

The open-label, single-arm, multicentre ORZORA trial (NCT02476968) evaluated maintenance olaparib in patients with platinum-sensitive relapsed ovarian cancer (PSR OC) with a germline (g) or somatic (s) *BRCA1* and/or *BRCA2* mutation (BRCAm) or a non-BRCA homologous recombination repair mutation (non-BRCA HRRm).

**Methods:**

Patients were in response to platinum-based chemotherapy after ≥2 prior lines of treatment and underwent prospective central screening for tumour BRCA status, then central gBRCAm testing to determine sBRCAm or gBRCAm status. An exploratory cohort evaluated non-BRCA HRRm in 13 predefined genes. Patients received olaparib 400 mg (capsules) twice daily until investigator-assessed disease progression. Secondary endpoints included overall survival (OS) and safety.

**Results:**

177 patients received olaparib. At the final data cutoff (25 June 2021), median OS from study enrolment was 46.8 (95% confidence interval [CI] 37.9–54.4), 43.2 (31.7–NC [not calculated]), 47.4 (37.9–NC) and 44.9 (28.9–NC) months in the BRCAm, sBRCAm, gBRCAm and non-BRCA HRRm cohorts, respectively. No new safety signals were identified.

**Conclusion:**

Maintenance olaparib showed consistent clinical activity in the BRCAm and sBRCAm cohorts; exploratory analysis suggested similar activity in the non-BRCA HRRm cohort. These findings highlight that patients with PSR OC, beyond those with gBRCAm, may benefit from maintenance olaparib.

## Introduction

Most patients with high-grade epithelial ovarian cancer typically respond to debulking surgery followed by platinum-based chemotherapy as a first-line treatment; however, around 70% of patients will eventually experience relapse [1]. Maintenance therapy with the poly(ADP-ribose) polymerase (PARP) inhibitor olaparib is a standard of care in platinum-sensitive relapsed ovarian cancer [2]. *BRCA1* and/or *BRCA2* mutations (BRCAm) are established mechanisms of homologous recombination deficiencies (HRDs) known to play a role in ovarian cancer and may be germline (g) or somatic (s) in origin. Approximately 20% of ovarian cancers have a BRCAm [3], with 15–17% of ovarian tumours reported to have a gBRCAm and 3–8% reported to have an sBRCAm [3, 4], although recent analysis of a large genomic dataset of ~75,000 tumour samples across six major tumour types suggested a 1:1 ratio of gBRCAm to sBRCAm in ovarian cancer [4].

Olaparib is a globally approved maintenance therapy for patients with platinum-sensitive relapsed ovarian cancer; in the USA olaparib is approved for patients with a BRCAm, whereas in Europe it is approved irrespective of BRCAm or other biomarker status [5–9]. Benefits of maintenance olaparib in patients with platinum-sensitive relapsed ovarian cancer have previously been reported. The Phase III SOLO2 trial demonstrated a clinically meaningful improvement in overall survival (OS) in patients with platinum-sensitive relapsed ovarian cancer and a gBRCAm receiving maintenance olaparib compared with placebo (hazard ratio [HR] 0.74; 95% confidence interval [CI] 0.54–1.00; *P* = 0.054). Although the inclusion criteria of the SOLO2 trial permitted enrolment of patients with an sBRCAm, no patients with an sBRCAm were enroled [10]. Furthermore, in the Phase II Study 19 trial, in patients with platinum-sensitive relapsed serous ovarian cancer, maintenance olaparib provided an apparent OS advantage compared with placebo regardless of BRCAm status (HR 0.73; 95% CI 0.55–0.95; nominal *P* = 0.021 in the full analysis set), although it did not meet the predefined threshold for statistical significance [11]. In Study 19, the OS Kaplan–Meier curves in the full analysis set, as well as in BRCAm and BRCA wildtype subgroups, separated in favour of olaparib as duration of follow-up increased, albeit without statistical significance being met [11]. This indicated the presence of a subset of patients experiencing long-term benefit from maintenance olaparib within the BRCA wildtype subgroup, suggestive of non-BRCA homologous recombination repair mutation (HRRm) [11].

Data evaluating the efficacy and safety of olaparib in patients with sBRCAm are limited [12, 13]; the ORZORA trial (NCT02476968) was designed to confirm the benefit of and provide rationale for the use of olaparib in this patient cohort and also included an exploratory cohort assessing maintenance olaparib in patients with a non-BRCA HRRm.

In the primary analysis of ORZORA (data cutoff [DCO] 17 April 2020), maintenance olaparib showed activity in patients with platinum-sensitive relapsed ovarian cancer and a BRCAm, including those with an sBRCAm [14]. Additionally, exploratory analyses showed activity in patients with a non-BRCA HRRm. Median progression-free survival (PFS) of 18.0 months (95% CI 14.3–22.1), 16.6 months (95% CI 12.4–22.2), 19.3 months (95% CI 14.3–27.6) and 16.4 months (95% CI 10.9–19.3) was reported in the BRCAm, sBRCAm, gBRCAm and non-BRCA HRRm cohorts, respectively [14].

In this analysis of the ORZORA trial, we report the final OS results alongside other secondary efficacy endpoints and updated safety and tolerability data.

## Methods

### Study design and patients

Study methods and full patient screening and enrolment criteria have previously been described in detail [14]. In brief, ORZORA was a Phase IV, open-label, single-arm, multicentre study of maintenance olaparib in patients with platinum-sensitive relapsed high-grade epithelial ovarian, primary peritoneal and/or fallopian tube cancer with a deleterious or suspected deleterious gBRCAm or sBRCAm, or a qualifying deleterious or suspected deleterious non-BRCA HRRm. Patients had platinum-sensitive disease, defined as disease progression ≥6 months after completion of the penultimate platinum-based chemotherapy regimen, and had completed ≥2 prior lines of platinum-based chemotherapy. Patients were in response, either complete or partial, following the last line of chemotherapy prior to study enrolment.

Patients were screened to determine BRCAm and HRRm status, undergoing prospective central screening for tumour BRCA status (MyChoice®CDx, Myriad Genetic Laboratories, Inc., Salt Lake City, UT, USA), then central gBRCAm testing (BRACAnalysis CDx®, Myriad Genetic Laboratories, Inc., Salt Lake City, UT, USA) to determine sBRCAm or gBRCAm status. Patients with previously confirmed gBRCAm were excluded to limit their inclusion and ensure ≥50 patients with sBRCAm were recruited. Following a protocol amendment (July 2016), patients without a tumour BRCAm were eligible for entry into the exploratory non-BRCA HRRm cohort. Non-BRCA HRRm status was determined via central tumour testing using the FoundationOne®CDx assay (Foundation Medicine, Inc., Cambridge, MA, USA). Qualifying genetic alterations were predicted deleterious or suspected deleterious loss of function mutations in any of the following 13 predefined genes involved (directly and indirectly) in the HRR pathway: *ATM*, *BARD1*, *BRIP1*, *CDK12*, *CHEK1*, *CHEK2*, *FANCL*, *PALB2*, *PPP2R2A*, *RAD51B*, *RAD51C*, *RAD51D* and *RAD54L*.

Within 8 weeks after their last dose of platinum-based chemotherapy, patients received maintenance olaparib capsules 400 mg twice daily (total daily dose: 800 mg) until investigator-assessed radiological disease progression (modified Response Evaluation Criteria in Solid Tumours [RECIST] version 1.1) or unacceptable toxicity. Some patients were permitted to continue olaparib beyond progression if the investigator deemed that the patient was benefitting from treatment in relation to other clinical assessments and did not meet other discontinuation criteria.

The trial was performed in accordance with the Declaration of Helsinki, Good Clinical Practice Guidelines and the AstraZeneca policy of bioethics [15] and was approved by the Ethics Committee of Istituto Nazionale Tumori ‘Fondazione G Pascale’ (Reference no. 25/15). All patients provided written informed consent.

### Endpoints and assessment

The co-primary endpoints of investigator-assessed PFS (modified RECIST version 1.1) in the BRCAm and sBRCAm cohorts have been reported previously [14].

OS and PFS2 were secondary endpoints; OS was defined as the time from date of study enrolment until death due to any cause and PFS2 was defined as the time to second progression or death. OS and PFS2 were reported in the any BRCAm and sBRCAm cohorts. Time to first subsequent therapy or death (TFST), time to second subsequent therapy or death (TSST), and safety and tolerability were also included in the secondary analyses in the any BRCAm and sBRCAm cohorts. OS and safety and tolerability were also assessed in the exploratory non-BRCA HRRm cohort. PFS2, TFST and TSST were not prespecified endpoints in the exploratory non-BRCA HRRm cohort.

The safety follow-up period was during study treatment and for 30 days after the last dose of olaparib. Myelodysplastic syndrome (MDS) events, acute myeloid leukaemia (AML) and new primary malignancy events were required to be reported even if they occurred after the end of the 30-day safety follow-up period.

### Statistical analysis

The DCO was 25 June 2021. OS was reported as Kaplan–Meier estimates in the BRCAm, sBRCAm, gBRCAm and non-BRCA HRRm cohorts. PFS2, TFST and TSST were reported as Kaplan–Meier estimates in the BRCAm, sBRCAm and gBRCAm cohorts.

Efficacy results were reported using the full analysis set, which included all patients who were assigned to olaparib treatment. Safety results were reported using the safety analysis set, which included all patients who received at least one dose of olaparib.

## Results

### Patients

**Fig. 1:**
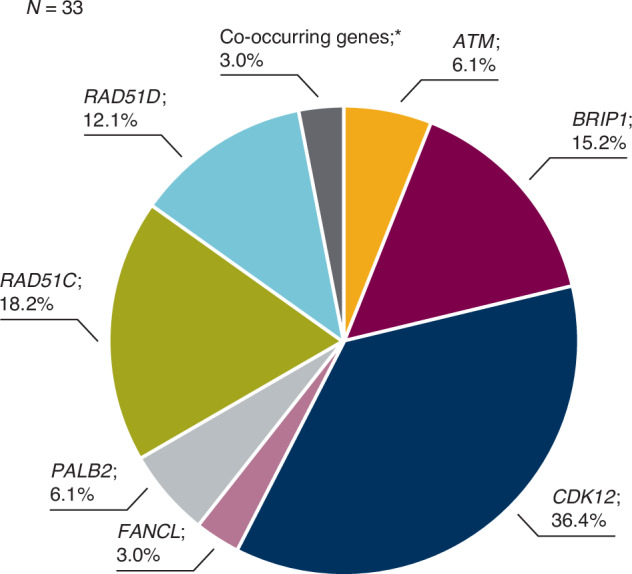
Frequency of non-BRCA HRR gene mutations in ORZORA [14]. In addition to the non-BRCA HRR gene mutations shown, non-BRCA HRR gene mutations with a prevalence of 0% included: *BARD1*, *CHEK1*, *CHEK2*, *PPP2R2A*, *RAD51B* and *RAD54L*. *Co-occurring genes included *CHEK2* and *RAD51C*. HRR homologous recombination repair.

Of the 872 patients screened, 181 were enroled into the ORZORA study and 177 received olaparib and were therefore included in the safety analyses. One hundred and forty-five patients were included in the BRCAm cohort, with 55 and 87 patients included in the sBRCAm and gBRCAm cohorts, respectively; three patients were assigned to the BRCAm cohort with unknown s/gBRCAm status. Three enroled patients had neither a BRCAm nor a non-BRCA HRRm and were unassigned. Thirty-three patients were included in the exploratory non-BRCA HRRm cohort (Supplementary Fig. S1); the breakdown of individual non-BRCA HRRm genes in ORZORA is shown in Fig. 1. Baseline characteristics have been reported previously [14] and were consistent across cohorts (Supplementary Table S1). At the final DCO (25 June 2021), 34 of 177 patients (19.2%) were still receiving maintenance olaparib, including 12 (21.8%) patients with an sBRCAm and 6 (18.8%) with a non-BRCA HRRm. One hundred and forty-three patients had discontinued olaparib (79.0%), for whom disease progression, observed in 102 patients (71.3%), was the most frequently reported reason for olaparib discontinuation (Supplementary Table S2).

### Overall survival

**Fig. 2:**
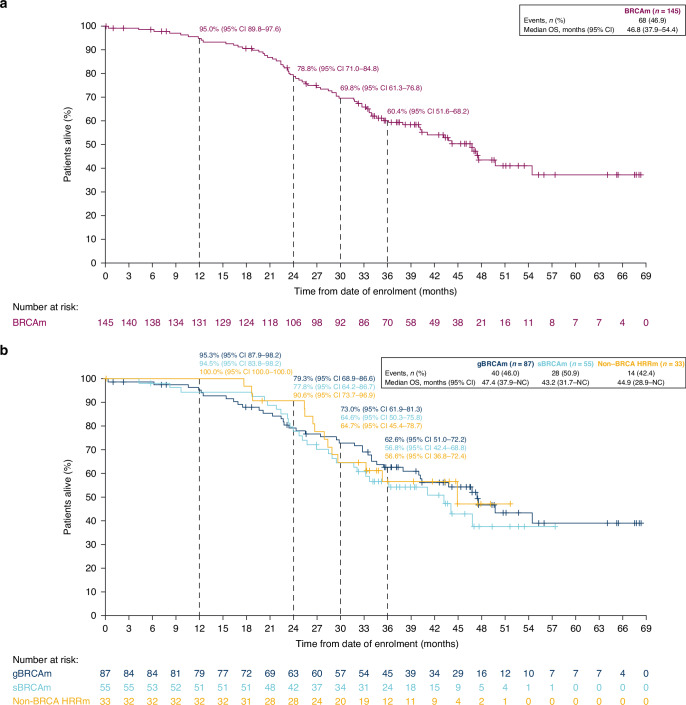
Kaplan–Meier plots of overall survival. Overall survival in patients with **a** BRCAm and **b** an sBRCAm, gBRCAm or non-BRCA HRRm. BRCAm *BRCA1* and/or *BRCA2* mutation, CI confidence interval, gBRCAm germline BRCAm, HRRm homologous recombination repair mutation, NC not calculated, OS overall survival, sBRCAm somatic BRCAm.

**Fig. 3:**
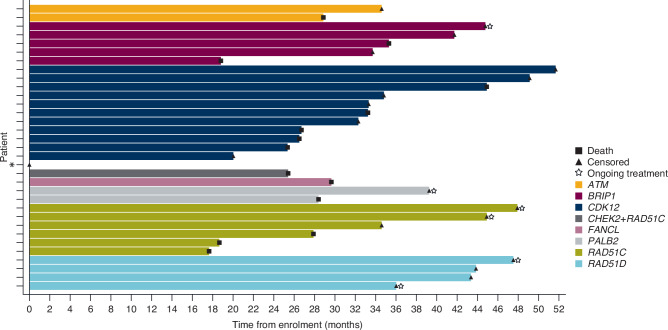
Swimmer plot of patient-level overall survival in the non-BRCA HRR mutation cohort. *Patient censored on Day 1 had a *CDK12* mutation. *HRR* homologous recombination repair.

The final OS analysis was performed after 68 of 145 patients had died (46.9% maturity) (BRCAm cohort), including 28 (50.9%) deaths in the sBRCAm cohort. In the non-BRCA HRRm cohort, 14 of 33 (42.4%) patients had died. The median duration of follow-up in patients censored for OS was 42.6 (range 0.03–68.3) months in the BRCAm cohort (39.5 [5.8–57.4] months in sBRCAm; 45.3 [1.0–68.3] months in gBRCAm) and 39.3 (0.03–51.7) months in the non-BRCA HRRm cohort. Median OS was consistent across all cohorts at more than 3.5 years. In the BRCAm, sBRCAm, gBRCAm and non-BRCA HRRm cohorts, median OS was 46.8 (95% CI 37.9–54.4), 43.2 (95% CI 31.7–NC [not calculated]), 47.4 (95% CI 37.9–NC) and 44.9 (95% CI 28.9–NC) months, respectively (Fig. 2). At 30 months, Kaplan–Meier estimates for OS rates were 69.8% (95% CI 61.3–76.8) in the BRCAm cohort, 64.6% (95% CI 50.3–75.8) in the sBRCAm cohort, 73.0% (95% CI 61.9–81.3) in the gBRCAm cohort and 64.7% (95% CI 45.4–78.7) in the non-BRCA HRRm cohort (Fig. 2). Kaplan–Meier estimates for 36-month OS rates were 60.4% (95% CI 51.6–68.2), 56.8% (95% CI 42.4–68.8), 62.6% (95% CI 51.0–72.2) and 56.6% (95% CI 36.8–72.4) in the BRCAm, sBRCAm, gBRCAm and exploratory non-BRCA HRRm cohort, respectively (Fig. 2). Patient-level OS varied across individual non-BRCA HRRm gene mutations (Fig. 3).

### Other secondary efficacy endpoints

At the time of final DCO, the maturity of PFS2 was 42.8% after 62 events in the BRCAm cohort. PFS2 was consistent across the BRCAm, sBRCAm and gBRCAm cohorts. At 30 months, the PFS2 rate was 58.1% (95% CI 48.1–66.8) in the BRCAm cohort, 48.7% (95% CI 32.9–62.8) in the sBRCAm cohort and 63.8% (95% CI 50.8–74.3) in the gBRCAm cohort (Kaplan–Meier estimates) (Supplementary Fig. S2A, B).

TFST analysis was performed at 50.3% maturity (BRCAm cohort). Consistent TFST results were observed in the BRCAm, sBRCAm and gBRCAm cohorts. At 30 months, the Kaplan–Meier estimate of the rate of freedom from the use of a first subsequent therapy or from death was 52.7% (95% CI 43.3–61.2) in the BRCAm cohort (51.2% [95% CI 36.3–64.3] in sBRCAm and 53.1% [95% CI 40.8–64.0] in gBRCAm) (Supplementary Fig. S3A, B). TSST was analysed at 41.4% maturity (BRCAm cohort); at 30 months, the Kaplan–Meier estimate of the rate of freedom from the use of a second subsequent therapy or from death was 61.1% (95% CI 51.3–69.4) in the BRCAm cohort (57.9% [95% CI 41.9–70.9] in sBRCAm and 62.6% [95% CI 49.9–73.0] in gBRCAm) (Kaplan–Meier estimates) (Supplementary Fig. S4A, B). The prespecified secondary efficacy endpoints of PFS2, TFST and TSST in the BRCAm, sBRCAm and gBRCAm cohorts are summarised in Supplementary Fig. S5. PFS2, TFST and TSST were not prespecified secondary endpoints in the exploratory non-BRCA HRRm cohort (data not shown).

Across all cohorts, patients received subsequent anticancer therapy; platinum-based chemotherapy was the most frequently reported subsequent therapy (Table 1). The proportion of patients receiving subsequent anticancer therapy was similar across the BRCAm cohorts.

**Table 1:** Subsequent anticancer therapy.

	Patient group, *n* (%)			
	BRCAm (*n* = 145)	gBRCAm (*n* = 87)	sBRCAm (*n* = 55)	Non-BRCA HRRm (*n* = 33)
Received subsequent anticancer therapy	59 (40.7)	35 (40.2)	23 (41.8)	18 (54.5)
Platinum-based chemotherapy^a^	48 (33.1)	29 (33.3)	18 (32.7)	15 (45.5)
Carboplatin	41 (28.3)	24 (27.6)	16 (29.1)	12 (36.4)
Cisplatin	11 (7.6)	8 (9.2)	3 (5.5)	5 (15.2)
Oxaliplatin	1 (0.7)	0 (0.0)	1 (1.8)	0 (0.0)
Paclitaxel	36 (24.8)	22 (25.3)	14 (25.5)	13 (39.4)
Gemcitabine	21 (14.5)	10 (11.5)	10 (18.2)	5 (15.2)
Anthracyclines and related substances	19 (13.1)	13 (14.9)	6 (10.9)	8 (24.2)
Doxorubicin^b^	18 (12.4)	12 (13.8)	6 (10.9)	8 (24.2)
Epirubicin hydrochloride	1 (0.7)	1 (1.1)	0 (0.0)	0 (0.0)
Other antineoplastic agents	10 (6.9)	9 (10.3)	0 (0.0)	2 (6.1)
Olaparib	5 (3.4)	4 (4.6)	0 (0.0)	0 (0.0)
Topotecan	4 (2.8)	4 (4.6)	0 (0.0)	2 (6.1)
Niraparib	1 (0.7)	1 (1.1)	0 (0.0)	1 (3.0)
Monoclonal antibodies	6 (4.1)	6 (6.9)	0 (0.0)	5 (15.2)
Anetumab ravtansine	1 (0.7)	1 (1.1)	0 (0.0)	0 (0.0)
Bevacizumab^c^	6 (4.1)	6 (6.9)	0 (0.0)	4 (12.1)
MBG 453	1 (0.7)	1 (1.1)	0 (0.0)	0 (0.0)
Pembrolizumab	0 (0.0)	0 (0.0)	0 (0.0)	1 (3.0)

*ATC* anatomical therapeutic chemical, *BRCAm BRCA1* and/or *BRCA2* mutation, *gBRCAm* germline BRCAm, *HRRm* homologous recombination repair mutation, *sBRCAm* somatic BRCAm.

Subsequent medications defined as medication taken after the end date of olaparib. ATC shown if received by ≥10% of patients in any cohort. Patients could receive one or more subsequent therapy.

^a^Patients received carboplatin, cisplatin or oxaliplatin in combination with a nonplatinum agent.

^b^Doxorubicin includes doxorubicin, doxorubicin hydrochloride, liposomal doxorubicin hydrochloride, pegylated liposomal doxorubicin and pegylated liposomal doxorubicin hydrochloride.

^c^Patients received bevacizumab in combination with chemotherapy (*n* = 8) or other agents (*n* = 2).

### Safety

The safety analyses included 177 patients and the median total treatment duration was 17.7 months (range 0.0–68.1 months). The adverse event (AE) profile was consistent with the primary analysis [14]. The majority of patients (93.8%) experienced at least one AE; however, most AEs were low-grade and only 11 patients (6.2%) had an AE leading to olaparib discontinuation at the final DCO. Treatment-emergent AEs leading to dose interruptions and reductions occurred in 91 patients (51.4%) and 51 patients (28.8%), respectively. The most common AEs of any grade included nausea (54.8% of patients), fatigue/asthenia (53.7%), anaemia (44.1%) and vomiting (28.2%). Common Terminology Criteria for Adverse Events (CTCAE) grade ≥3 AEs were reported in 37.9% of patients and the most common grade ≥3 AE was anaemia, occurring in 16.4% of patients across the cohorts (Supplementary Table S3). Serious AEs were reported in 48 patients (27.1%) and AEs with an outcome of death were reported in 4 patients (2.3%). Neutropenia, diarrhoea, AML and MDS each led to discontinuation of olaparib in two patients (1.1%) (Supplementary Table S4).

Since the primary DCO (17 April 2020), MDS was reported in four patients (2.3%; in whom two cases of MDS were reported after the last dose of olaparib) and no new cases of AML were reported (two cases of AML were reported at the primary DCO [14]). Therefore, a total of six patients (3.4%) had experienced MDS/AML events at the final DCO (25 June 2021). One patient (0.6%) had experienced a new primary malignancy since the primary DCO, non-Hodgkin lymphoma. The total incidence of new primary malignancies was low; three patients (1.7%) experienced a new primary malignancy in the ORZORA study at the final DCO (one case of Burkitt’s lymphoma and one case of papillary thyroid cancer were reported at the primary DCO [14]).

## Discussion

In the Phase IV ORZORA trial, maintenance olaparib for patients with platinum-sensitive relapsed ovarian cancer, who were in response to their most recent platinum-based chemotherapy after ≥2 prior lines of platinum-based chemotherapy, demonstrated consistent clinical activity in patients with a BRCAm, irrespective of germline or somatic status, or a non-BRCA HRRm. The primary analysis demonstrated the clinical activity of maintenance olaparib in patients with platinum-sensitive relapsed ovarian cancer and an sBRCAm and expanded on the limited data available for this patient cohort [14]. The final OS analysis reported here further defined the clinical activity of maintenance olaparib for patients with platinum-sensitive relapsed ovarian cancer, beyond those with a gBRCAm, with consistent OS and supportive clinical efficacy results reported across the different patient cohorts.

In the ORZORA study, median OS data reported in the BRCAm (46.8 months), sBRCAm (43.2 months), gBRCAm (47.4 months) and non-BRCA HRRm (44.9 months) cohorts were consistent with results reported in patients with platinum-sensitive relapsed ovarian cancer and a gBRCAm receiving maintenance olaparib in the SOLO2 trial (median OS 51.7 months vs. 38.8 months with placebo [HR 0.74; 95% CI 0.54–1.00; *P* = 0.054]) [10]. In the OS analysis of the Study 19 trial, maintenance olaparib provided a trend in OS compared with placebo, with median OS of 29.8 months in the olaparib arm and 27.8 months in the placebo arm (full analysis set; HR 0.73; 95% CI 0.55–0.95; nominal *P* = 0.021); however, the predefined threshold of statistical significance was not met [11]. Additionally, in the single-arm OPINION trial, the first study to prospectively assess maintenance olaparib monotherapy in patients with platinum-sensitive relapsed ovarian cancer without a gBRCAm, median OS of 32.7 months was reported for patients receiving maintenance olaparib [13, 16]. OPINION included a small group of patients with confirmed sBRCAm status; the 30-month Kaplan–Meier OS rate was 70.4% (95% CI 49.4–83.9) [16]. The OS results reported in the ORZORA trial may provide supportive evidence for the use of maintenance olaparib in patients with platinum-sensitive relapsed ovarian cancer and an sBRCAm or non-BRCA HRRm. In the newly diagnosed ovarian cancer setting, olaparib is approved for use as a first-line maintenance treatment. Maintenance olaparib improved OS versus placebo in patients with a BRCAm after 7 years of follow-up in the SOLO1 study (HR 0.55; 95% CI 0.40–0.76; *P* = 0.0004 [*P* < 0.0001 required to declare statistical significance]) [17], and maintenance olaparib plus bevacizumab provided a clinically meaningful OS improvement versus placebo plus bevacizumab in patients who tested HRD-positive after 5 years of follow-up in the PAOLA-1 study (HR 0.62; 95% CI 0.45–0.85) [18].

While comparisons between ORZORA and Phase III randomised controlled trials such as NOVA and ARIEL3 should be avoided due to differences in study design and patient populations, it is worth noting that although a PFS benefit was demonstrated in the primary analysis of NOVA, which evaluated maintenance niraparib versus placebo in patients with platinum-sensitive relapsed ovarian cancer with or without a gBRCAm [19], no OS benefit was seen in the non-gBRCAm cohort (including HRD-positive non-gBRCAm) who received niraparib [20, 21]. Similarly, although a PFS benefit was demonstrated in the primary analysis of ARIEL3, which evaluated maintenance rucaparib versus placebo in the relapsed disease setting [22], no OS benefit was observed in the HRD-positive cohort [23, 24]. These studies were not statistically powered for OS and crossover confounded these results; thus, interpretation is limited [23, 24]. However, these OS results from NOVA and ARIEL3 have led to restriction of maintenance niraparib to patients with gBRCAm platinum-sensitive relapsed ovarian cancer [25] and maintenance rucaparib to patients with BRCAm platinum-sensitive relapsed ovarian cancer [26] in the USA. Moreover, based on these data from NOVA and ARIEL3, maintenance olaparib was restricted to patients with BRCAm platinum-sensitive relapsed ovarian cancer in the USA [7].

Interestingly, in ORZORA, the exploratory non-BRCA HRRm analyses suggest a similar level of activity in patients with a non-BRCA HRRm to that seen in patients with BRCAm. Exploratory subgroup analyses from Study 19 of patients with platinum-sensitive relapsed ovarian cancer and a non-BRCA HRRm (*n* = 21) suggested a PFS benefit for maintenance olaparib versus placebo with an HR of 0.21 (95% CI 0.04–0.86) (HR for OS of 0.77 [95% CI 0.28–2.28]) compared with patients without a BRCAm or HRRm (*n* = 58) (HR for PFS of 0.71 [95% CI 0.37–1.35]; HR for OS of 1.19 [95% CI 0.66–2.10]) [27]. However, the small number of patients with a non-BRCA HRRm limits the interpretation of these results. Exploratory analyses from NOVA and ARIEL3 also suggested a PFS benefit in the subgroup of patients with non-BRCA HRRm (HR for PFS with maintenance niraparib vs. placebo of 0.31 [95% CI 0.13–0.77], *n* = 41 [28] and for maintenance rucaparib vs. placebo of 0.21 [95% CI 0.09–0.50], *n* = 43 [29]), although small patient numbers limit interpretation of these data. By contrast, non-BRCA HRRm gene panels were not predictive of PFS benefit with maintenance olaparib plus bevacizumab in the newly diagnosed advanced ovarian cancer setting in the PAOLA-1 study [30].

In the ORZORA study, 40.7% of patients in the BRCAm cohort received a subsequent anticancer therapy; similar levels of subsequent anticancer therapy were reported in the other patient cohorts. These data are important for the contextualisation of the OS results. Assessing improvements in OS can prove difficult in ovarian cancer trials as patients often receive multiple subsequent anticancer therapies post-progression, including experimental agents [31, 32]. Moreover, mortality unrelated to cancer or the treatment under investigation can confound OS results. Therefore, evaluation of supportive efficacy endpoints is useful to provide a comprehensive evaluation of efficacy and could be considered an appropriate surrogate for OS [32]. The supportive efficacy endpoints (PFS2, TFST and TSST) reported in this analysis are comparable with values published for SOLO2 and Study 19 [10, 11]. PFS2, TFST and TSST also favoured PARP inhibitor maintenance over placebo in NOVA and ARIEL3 [33, 34].

The safety profile of maintenance olaparib was consistent with the primary analysis [14] and with previous reports in this setting [10, 16], and no new safety findings were identified. Consistent with previous PARP inhibitor studies, AEs in ORZORA were usually managed by dose interruption [35]; dose interruptions due to AEs were reported in 51.4% of patients. In line with the primary analysis, the incidence of MDS/AML remained low, with a total incidence of 3.4% at the final DCO. An imbalance in MDS/AML was observed between maintenance olaparib and placebo (8% vs. 4% of patients) after approximately 5 years’ follow-up in the relapsed disease setting in SOLO2 [10]. Factors that may have contributed to this imbalance include potential baseline risk factors, MDS/AML being a long latency event and the longer duration of OS observed in the olaparib arm. The incidence of MDS/AML remained low with longer-term follow-up in the newly diagnosed ovarian cancer setting. In SOLO1, the incidence of MDS/AML after 7 years of follow-up was 1.5% in the maintenance olaparib group and 0.8% in the placebo group [17]. In PAOLA-1, the incidence of MDS/AML/aplastic anaemia after 5 years of follow-up was 1.7% in the olaparib plus bevacizumab group and 2.2% in the placebo plus bevacizumab group [18]. The incidence of new primary malignancies was low (1.7%) at the final DCO in the ORZORA trial.

The limitations of the ORZORA trial have been described fully by Pignata et al. and include the lack of comparator arm [14]; however, given the benefit of maintenance olaparib, a comparator arm was deemed inappropriate. Moreover, the recent succession of olaparib capsules by olaparib tablets resulted in early termination of the study due to discontinuation of olaparib capsules prior to the pre-planned final OS analysis at 60% maturity (OS results reported here at 46.9% maturity). The small size and heterogeneity of the exploratory non-BRCA HRRm cohort limits the ability to assess the predictive benefit of olaparib monotherapy, which may be associated with individual non-BRCA HRR genes. Finally, the supportive efficacy endpoints reported here have methodological limitations, with PFS2 also affected by subsequent therapy and TSFT and TSST influenced by the investigator and patient [32]. However, the inclusion of intermediate clinical endpoints, including PFS2, TFST and TSST, can further define efficacy and provide supportive evidence for the clinical activity of maintenance olaparib demonstrated by PFS and OS [32].

OS results reported from the final analysis continue to demonstrate the clinical activity of maintenance olaparib in patients with platinum-sensitive relapsed ovarian cancer with an sBRCAm or BRCAm. Exploratory OS analyses suggest similar olaparib activity can be achieved in patients with platinum-sensitive relapsed ovarian cancer and a non-BRCA HRRm compared with patients with a BRCAm. The safety profile observed was consistent with previous studies of maintenance olaparib in the relapsed disease setting and the primary analysis, with no new safety findings identified. Taken together, these results highlight that patients with platinum-sensitive relapsed ovarian cancer, beyond those with a gBRCAm, may benefit from maintenance olaparib.

## Supplementary information

Online Supplementary Appendix to: Overall survival with maintenance olaparib in platinum-sensitive relapsed ovarian cancer by somatic or germline BRCA and homologous recombination repair mutation stat

## Data Availability

Data underlying the findings described in this manuscript may be obtained in accordance with AstraZeneca’s data sharing policy described at https://astrazenecagrouptrials.pharmacm.com/ST/Submission/Disclosure. Data for studies directly listed on Vivli can be requested through Vivli at www.vivli.org. Data for studies not listed on Vivli could be requested through Vivli at https://vivli.org/members/enquiries-about-studies-not-listed-on-the-vivli-platform/. AstraZeneca Vivli member page is also available outlining further details: https://vivli.org/ourmember/astrazeneca/.
