# Supplementary material for: Overall survival with maintenance olaparib in platinum-sensitive relapsed ovarian cancer by somatic or germline BRCA and homologous recombination repair mutation status
Source: Br J Cancer. 2025 Mar 17;132(8):725–32. doi: 10.1038/s41416-025-02966-x (PMC11997082; doi:10.1038/s41416-025-02966-x)
Supplement: Supplementary file 1 — Online Supplementary Appendix to: Overall survival with maintenance olaparib in platinum-sensitive relapsed ovarian cancer by somatic or germline BRCA and homologous recombination repair mutation stat [file 41416_2025_2966_MOESM1_ESM.pdf]

ONLINE SUPPLEMENTARY APPENDIX TO:

Overall survival with maintenance olaparib in platinum-sensitive relapsed ovarian cancer by somatic or germline BRCA and homologous recombination repair mutation status

Pignata S, Oza A, Hall G et al.
Email: s.pignata@istitutotumori.na.it

ORZORA investigators

The principal investigators for each site that participated in the study are shown below.

Country	Principal investigators
Bulgaria	Zhanet Grudeva-Popova, Krassimir Orechkov, Tatyana Koynova, Antoaneta Tomova, Rumyana Ilieva, Constanta Timcheva, Galina Kurteva, Ivan Donev, Koynov Krassimir
Canada	Stephan Welch, Amit Oza, Prafull Ghatage, Diane Provencher, James Bentley, Susie Lau
Czech Republic	Zdenek Kral, Barbara Donocikova, Bohuslav Melichar, David Cibula, Jana Prausova, Maria Zvarikova, Jaroslav Klat
Hungary	Zsuzsanna Papai, Katalin Boer, Robert Poka, Gabor Pajkos, Imre Pete, Gyorgy Bodoky
Italy	Sandro Pignata, Nicoletta Colombo, Anna Maria Mosconi, Gennaro Cormio, Giampaolo Tortora
Poland	Dariusz Wydra, Radoslaw Madry, Ewa Nowak-Markwitz, Beata Mackowiak-Matejczyk, Marcin Misiek
Spain	Beatriz Pardo, David Vicente Baz, Antonio Casado Herraez, Cristina Martin Lorente, Ana Santaballa Bertran, Jose Alejandro Perez Fidalgo, Cesar Mendiola, Enrique Lastra Aras, Juana Oramas, Margarita Romeo, Ana Herrero
United Kingdom	Geoff Hall, Shibani Nicum, Emma Hudson, Rachel Jones, Emma Cattell, Yvette Drew, Hafiz Algurafi, Andrew Clamp, Joanne Millar, Sarah Williams, Rosemary Lord, Ana Montes Borinaga, Rosalind Glasspool

STUDY DESIGN AND PATIENTS

Patients underwent prospective central screening for tumour (t) BRCA status (MyChoice® CDx, Myriad Genetic Laboratories, Inc., Salt Lake City, UT, USA). tBRCA mutations included either somatic (s) or germline (g) BRCA mutations. To further define sBRCA and gBRCA mutation status, patients underwent central gBRCA mutation testing using the BRACAnalysis CDx assay (Myriad Genetic Laboratories, Inc). Those with sBRCA mutations were defined as being tBRCA mutation-positive and gBRCA mutation-negative.

Patients received maintenance olaparib until investigator-assessed objective radiological disease progression (objective tumour response was assessed according to Response Evaluation Criteria in Solid Tumours [RECIST v1.1] criteria) or as long as, in the investigator’s opinion, they were benefitting from treatment in relation to other clinical assessments and they did not meet any other discontinuation criteria. Patients were permitted to discontinue investigational treatment for the following reasons besides disease progression: patient decision; adverse event; bone marrow findings consistent with myelodysplastic syndrome or acute myeloid leukaemia; or severe noncompliance with the study protocol.

Table S1. Patient demographic and disease characteristics at baseline (full analysis set) [1].

Characteristic	BRCAm[a] (*n* = 145)	gBRCAm (*n* = 87)	sBRCAm (*n* = 55)	Non-BRCA HRRm (*n* = 33)
Patient age, years, median (range)[b]	61.5 (39–82)	56.0 (39–82)	67.0 (42–78)	64.0 (45–79)
Time from original diagnosis, years, median (range)	3.05 (1.4–25.3)	3.37 (1.4–15.3)	2.93 (1.5–25.3)	3.52 (1.7–9.4)
Primary tumour location, *n* (%)				
Ovary	124 (86)	78 (90)	43 (78)	27 (82)
Fallopian tubes	7 (5)	2 (2)	5 (9)	1 (3)
Primary peritoneal	14 (10)	7 (8)	7 (13)	5 (15)
Histology, *n* (%)				
Serous	131 (90)	76 (87)	52 (95)	29 (88)
Mucinous	2 (1)	1 (1)	1 (2)	0 (0)
Clear cell	2 (1)	2 (2)	0 (0)	2 (6)
Endometrioid	3 (2)	3 (3)	0 (0)	1 (3)
Undifferentiated	5 (3)	4 (5)	1 (2)	0 (0)
Mixed, epithelial	2 (1)	1 (1)	1 (2)	1 (3)
Prior lines of chemotherapy, *n* (%)				
2	80 (55)	45 (52)	34 (62)	18 (55)
3	41 (28)	28 (32)	12 (22)	11 (33)
≥4	23 (16)	14 (16)	9 (16)	4 (12)
Missing	1 (1)	0 (0)	0 (0)	0 (0)
Response to previous platinum-based chemotherapy, *n* (%)[c]				
Complete response	75 (52)	44 (51)	30 (55)	11 (33)
Partial response	68 (47)	43 (49)	25 (45)	21 (64)
Missing	2 (1)	0 (0)	0 (0)	1 (3)

Characteristic	BRCAm[a] (*n* = 145)	gBRCAm (*n* = 87)	sBRCAm (*n* = 55)	Non-BRCA HRRm (*n* = 33)
tBRCAm, *n* (%)[d]	124 (100)	66 (100)	55 (100)	0 (0)
BRCA1	81 (65)	42 (64)	36 (65)	0 (0)
BRCA2	42 (34)	23 (35)	19 (35)	0 (0)
Both	1 (1)	1 (2)	0 (0)	0 (0)

BRCAm BRCA1 and/or *BRCA2* mutation, *FIGO* International Federation of Gynecology and Obstetrics, *g* germline, *HRRm* homologous recombination repair mutation, *s* somatic, *t* tumour.

[a]BRCAm cohort includes three patients who reported a BRCAm but could not be classified as sBRCAm or gBRCAm.

[b]Patients for whom only year of birth was recorded (due to country restrictions) are excluded from this summary.

[c]Response to previous platinum-based chemotherapy immediately prior to enrolment into the study.

[d]Local or Myriad testing.

Table S2. Patient disposition.

	BRCAm	gBRCAm	sBRCAm	Non-BRCA HRRm	Total
Patients screened, *n*	167	97	67	42	872[a]
Patients enrolled (FAS), *n*	145[b]	87	55	33	181[c]
Treated (SAS), *n* (%)	143 (98.6)	87 (100)	55 (100)	32 (97.0)	177[d]
Ongoing study treatment at OS DCO, *n* (%)[e]	28 (19.3)	16 (18.4)	12 (21.8)	6 (18.2)	34 (18.8)
Discontinued study treatment, *n* (%)[e]	115 (79.3)	71 (81.6)	43 (78.2)	26 (78.8)	143 (79.0)
Patient decision	16 (13.9)	10 (14.1)	6 (14.0)	2 (7.7)	18 (12.6)
Adverse event	10 (8.7)	7 (9.9)	3 (7.0)	1 (3.8)	11 (7.7)
Severe non-compliance to protocol	0 (0.0)	0 (0.0)	0 (0.0)	0 (0.0)	0 (0.0)
Condition under investigation worsened	81 (70.4)	49 (69.0)	31 (72.1)	20 (76.9)	102 (71.3)
Development of study-specific discontinuation criteria	2 (1.7)	1 (1.4)	1 (2.3)	0 (0.0)	2 (1.4)
Patient lost to follow-up	0 (0.0)	0 (0.0)	0 (0.0)	0 (0.0)	0 (0.0)
Other	6 (5.2)	4 (5.6)	2 (4.7)	3 (11.5)	10 (7.0)

BRCAm BRCA1 and/or *BRCA2* mutation, *DCO* data cutoff, *FAS* full analysis set, *g* germline, *HRRm* homologous recombination repair mutation, *OS* overall survival, *s* somatic, *SAS* statistical analysis set.

[a]Includes 663 unassigned patients without a BRCAm or non-BRCA HRRm.

[b]BRCAm cohort includes three patients who reported a BRCAm but could not be classified as sBRCAm or gBRCAm.

[c]Includes three unassigned patients without a BRCAm or non-BRCA HRRm.

[d]ncludes two of the three unassigned patients (neither of these patients were receiving ongoing study treatment at the final data cut-off).

[e]Percentage calculated from patients in the FAS with study treatment discontinuation and no missing data.

Table S3. Safety summary.

AEs	Patients, *n* (%) (*N* = 177)	
	**Any grade**	**Grade ≥3**
Any	166 (93.8)	67 (37.9)
Nausea	97 (54.8)	2 (1.1)
Fatigue/asthenia[a]	95 (53.7)	4 (2.3)
Anaemia[b]	78 (44.1)	29 (16.4)
Vomiting	50 (28.2)	2 (1.1)
Diarrhoea	32 (18.1)	2 (1.1)
Abdominal pain	31 (17.5)	1 (0.6)
Dyspepsia	28 (15.8)	0 (0.0)
Neutropenia[c]	26 (14.7)	3 (1.7)
Decreased appetite	22 (12.4)	1 (0.6)
Headache	20 (11.3)	0 (0.0)
Dizziness	20 (11.3)	0 (0.0)
Cough	20 (11.3)	0 (0.0)
Dyspnoea	20 (11.3)	1 (0.6)
Thrombocytopenia[d]	19 (10.7)	4 (2.3)
Constipation	18 (10.2)	0 (0.0)

AE adverse event.

Data are shown for any AE that occurred in at least 10% of patients and grade ≥3 AEs are reported for these most common AEs. AEs were graded using the Common Terminology Criteria for Adverse Events (version 4.0).

[a]Grouped term includes fatigue and asthenia.

[b]Grouped term includes anaemia, macrocytic anaemia, erythropenia, decreased haematocrit, decreased haemoglobin, normochromic anaemia, normochromic normocytic anaemia, normocytic anaemia and decreased red blood cell count.

[c]Grouped term includes agranulocytosis, febrile neutropenia, decreased granulocyte count, granulocytopenia, idiopathic neutropenia, neutropenia, neutropenic infection, neutropenic sepsis and decreased neutrophil count.

[d]Grouped term includes decreased platelet count, decreased platelet production, decreased plateletcrit, thrombocytopenia.

Table S4. Summary of AEs leading to olaparib discontinuation.

AE leading to discontinuation	Patients, *n* (%) (*N* = 177)
Any	11 (6.2)
AML	2 (1.1)
MDS[a]	2 (1.1)
Neutropenia[b]	2 (1.1)
Diarrhoea	2 (1.1)
Anaemia[c]	1 (0.6)
Thrombocytopenia[d]	1 (0.6)
Non-Hodgkin lymphoma	1 (0.6)
Small intestine obstruction	1 (0.6)
Fatigue/asthenia[e]	1 (0.6)
Anxiety	1 (0.6)

AE adverse event, *AML* acute myeloid leukaemia, *MDS* myelodysplastic syndrome.

[a]Reported in a total of four patients, in whom two cases of MDS were reported after the last dose of olaparib.

[b]Grouped term includes agranulocytosis, febrile neutropenia, decreased granulocyte count, granulocytopenia, idiopathic neutropenia, neutropenia, neutropenic infection, neutropenic sepsis and decreased neutrophil count.

[c]Grouped term includes anaemia, macrocytic anaemia, erythropenia, decreased haematocrit, decreased haemoglobin, normochromic anaemia, normochromic normocytic anaemia, normocytic anaemia and decreased red blood cell count.

[d]Grouped term includes decreased platelet count, decreased platelet production, decreased plateletcrit, thrombocytopenia.

[e]Grouped term includes fatigue and asthenia.

Figure S1. Patient assignment to cohorts in the ORZORA study

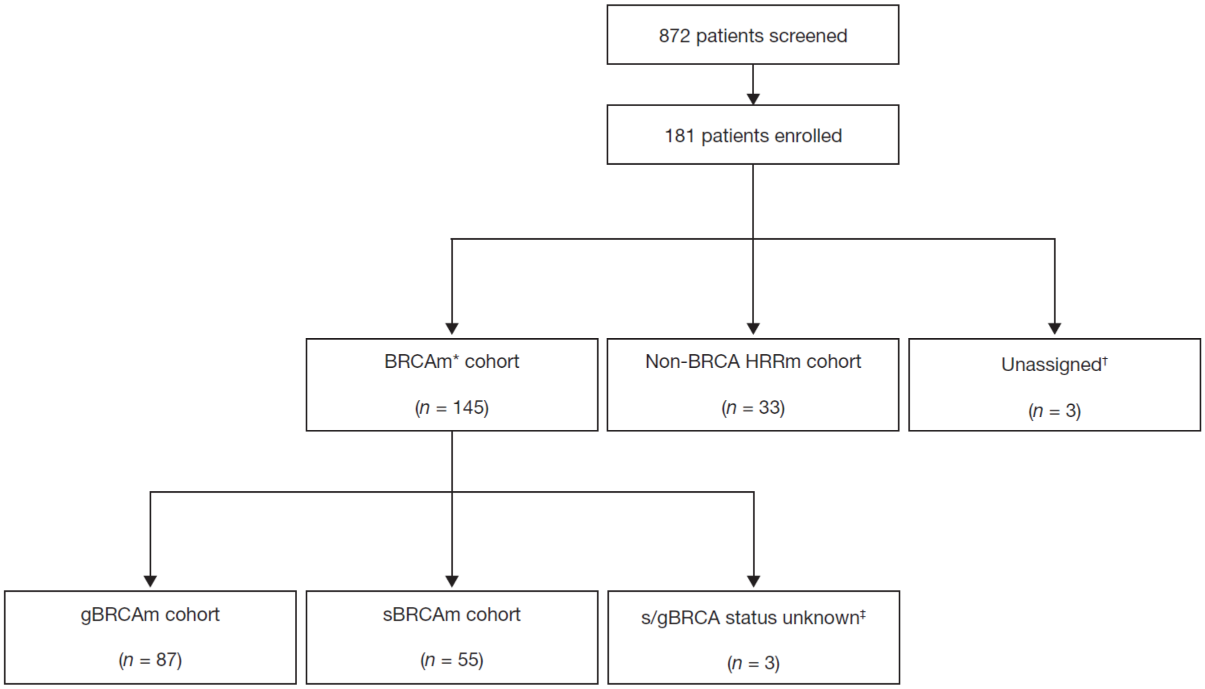

*Based on tumour BRCA status determined by prospective central screening using the Myriad MyChoice® CDx assay; †Unassigned patients were patients without a BRCAm or non-BRCA HRRm; ‡Three patients had a BRCAm but could not be classified as sBRCAm or gBRCAm.

BRCAm BRCA1 and/or *BRCA2* mutation, *g* germline, *HRRm* homologous recombination repair mutation, *s* somatic.

Figure S2. Kaplan–Meier plots of PFS2 in patients with BRCAm (**A**), and an sBRCAm or a gBRCAm (**B**)

A

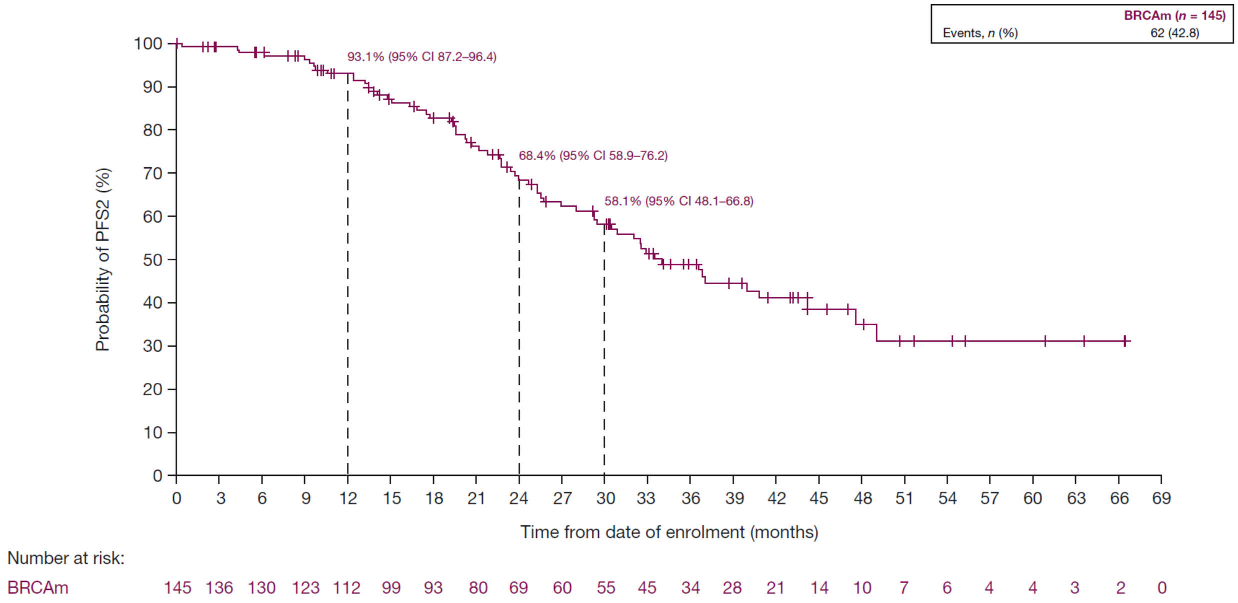

B

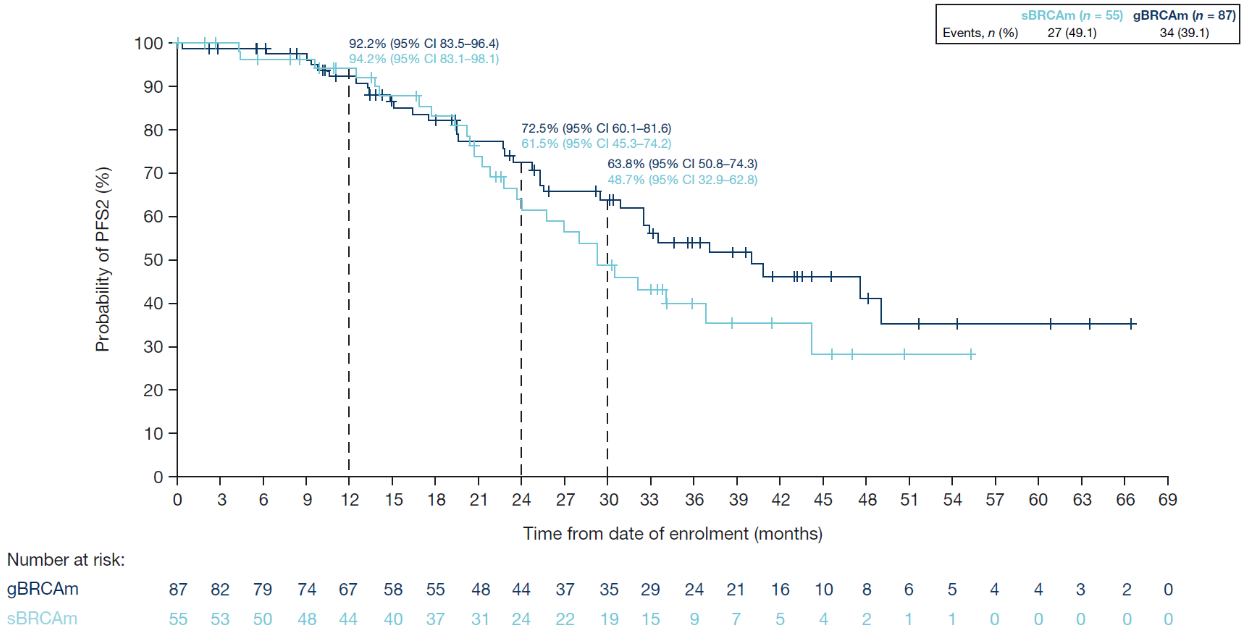

Medians for PFS2 are not reported due to lack of data maturity.

BRCAm BRCA1 and/or *BRCA2* mutation, *CI* confidence interval, *gBRCAm* germline BRCAm, *sBRCAm* somatic BRCAm, *PFS2* time to second progression or death.

Figure S3. Kaplan–Meier plots of TFST in patients with BRCAm (**A**), and an sBRCAm or a gBRCAm (**B**)

A

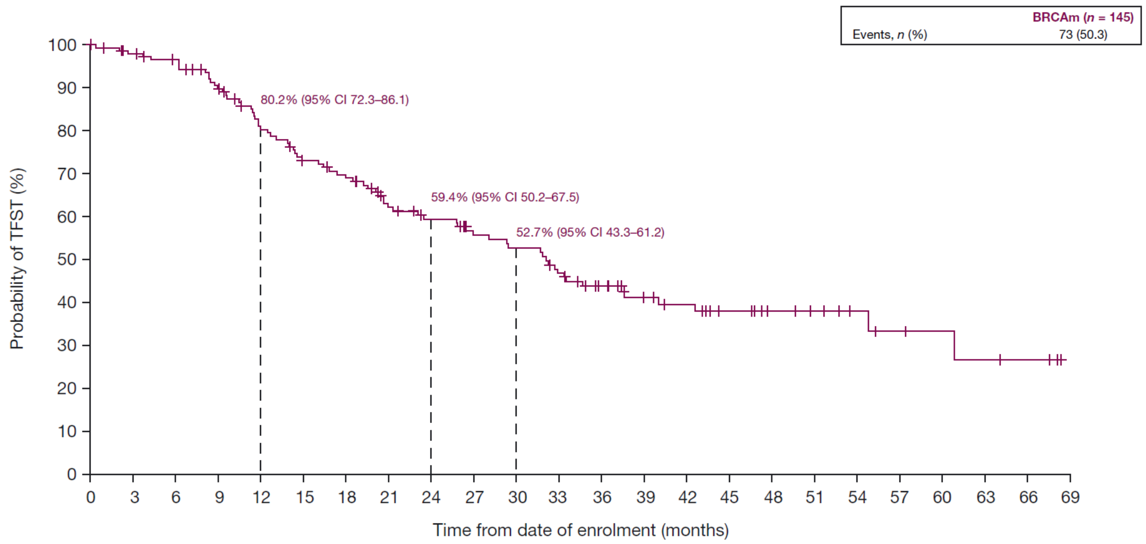

Number at risk:

	0	3	6	9	12	15	18	21	24	27	30	33	36	39	42	45	48	51	54	57	60	63	66	69
BRCAm	145	137	131	118	101	90	84	71	64	57	54	47	39	29	25	18	13	11	8	6	5	4	3	0

B

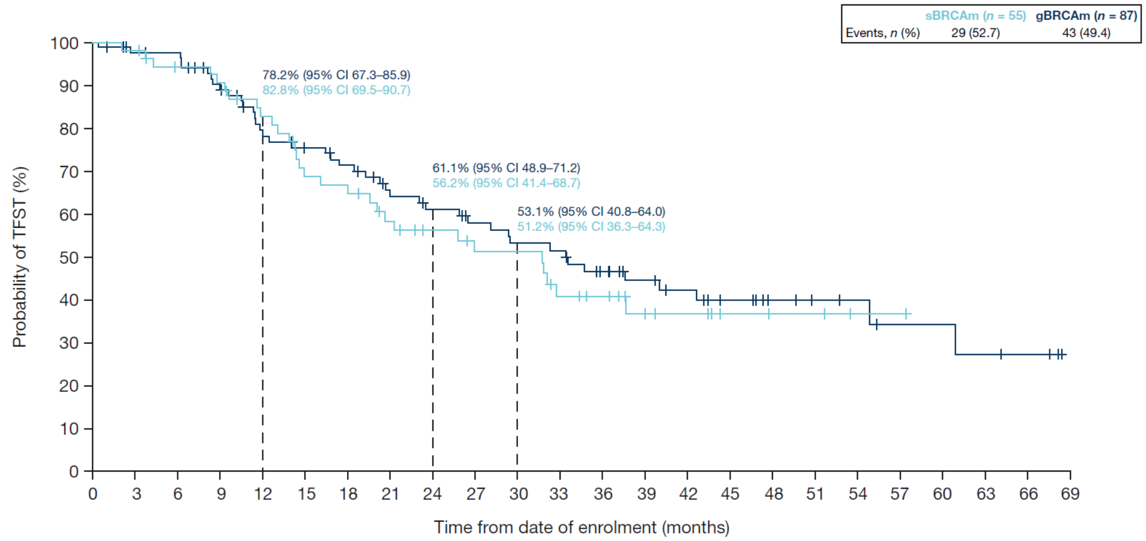

Number at risk:

	0	3	6	9	12	15	18	21	24	27	30	33	36	39	42	45	48	51	54	57	60	63	66	69
gBRCAm	87	82	81	70	58	55	51	43	40	36	33	32	26	21	18	14	10	8	7	5	5	4	3	0
sBRCAm	55	54	49	47	42	34	32	27	23	20	20	15	13	8	7	4	3	3	1	1	0	0	0	0

Medians for TFST are not reported due to lack of data maturity.

BRCAm BRCA1 and/or *BRCA2* mutation, *CI* confidence interval, *gBRCAm* germline BRCAm, *sBRCAm* somatic BRCAm, *TFST* time to first subsequent therapy or death.

Figure S4. Kaplan–Meier plots of TSST in patients with BRCAm (**A**), and an sBRCAm or a gBRCAm (**B**)

A

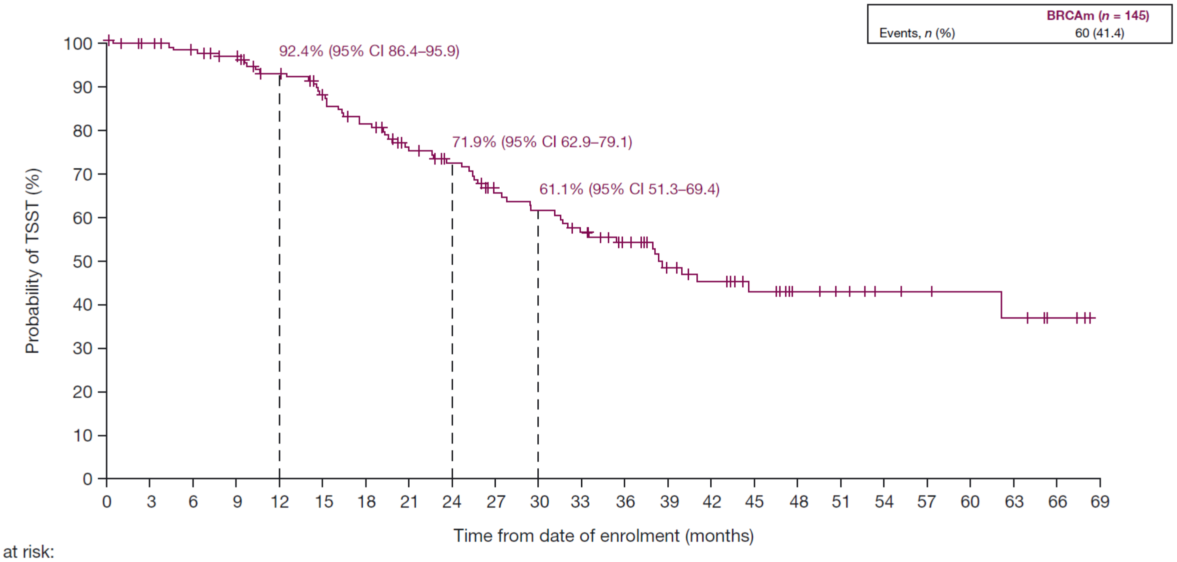

Number at risk:

BRCAm	145	139	133	127	115	105	96	83	75	64	60	54	45	32	27	20	14	12	9	8	7	6	3	0

B

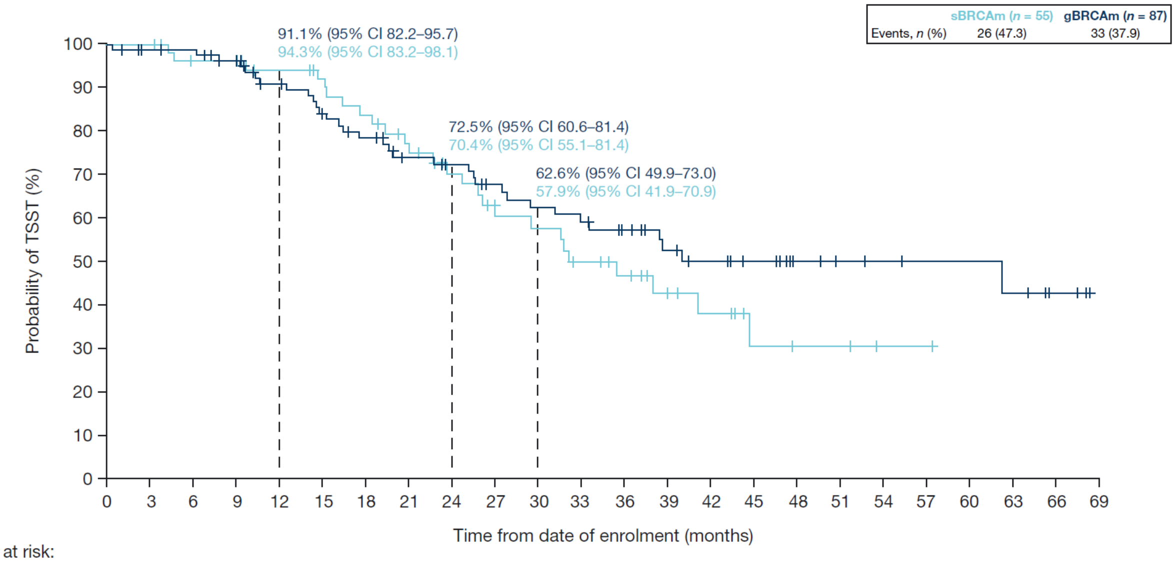

Number at risk:

gBRCAm	87	83	82	76	67	60	55	48	45	40	37	35	29	22	19	16	11	9	8	7	7	6	3	0
sBRCAm	55	55	50	50	47	44	40	34	29	23	22	18	15	10	8	4	3	3	1	1	0	0	0	0

Medians for TSST are not reported due to lack of data maturity.

BRCAm BRCA1 and/or *BRCA2* mutation, *CI* confidence interval, *gBRCAm* germline BRCAm, *sBRCAm* somatic BRCAm, TSST time to second subsequent therapy or death.

Figure S5. Summary of secondary efficacy endpoints in patients with a BRCAm (**A**), sBRCAm (**B**) and gBRCAm (**C**)

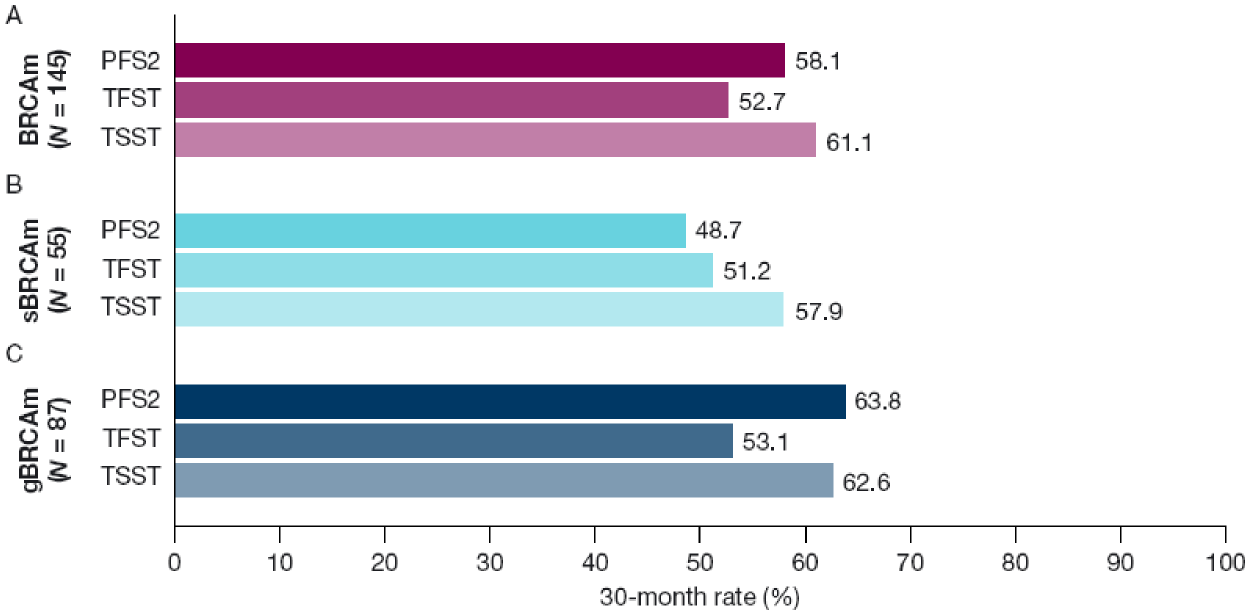

BRCAm BRCA1 and/or *BRCA2* mutation, *gBRCAm* germline BRCAm, *PFS2,* time to second progression or death, *sBRCAm* somatic BRCAm. *TFST* time to first subsequent therapy or death, *TSST* time to second subsequent therapy or death.

REFERENCE

1. Pignata S, Oza A, Hall G, Pardo B, Madry R, Cibula D, et al. Maintenance olaparib in patients with platinum-sensitive relapsed ovarian cancer: Outcomes by somatic and germline BRCA and other homologous recombination repair gene mutation status in the ORZORA trial. Gynecol Oncol. 2023;172:121-9.
